# A high-throughput structural system biology approach to increase structure representation of proteins from *Clostridioides difficile*

**DOI:** 10.1128/MRA.00507-23

**Published:** 2023-09-25

**Authors:** Monica Rosas-Lemus, Supratim Dey, George Minasov, Kemin Tan, Spencer M. Anderson, Joseph Brunzelle, Salvatore Nocadello, Ivan Shabalin, Ekaterina Filippova, Andrei Halavaty, Youngchang Kim, Natalia Maltseva, Jerzy Osipiuk, Wladek Minor, Andrzej Joachimiak, Alexei Savchenko, Wayne F. Anderson, Karla J. F. Satchell, Center for Structural Biology of Infectious Diseases team members

**Affiliations:** 1 Department of Microbiology-Immunology, Feinberg School of Medicine, Northwestern University, Chicago, Illinois, USA; 2 Center for Structural Biology of Infectious Diseases, Chicago, Illinois, USA; 3 Consortium for Advanced Science and Engineering, University of Chicago, Chicago, Illinois, USA; 4 Structural Biology Center, X-ray Science Division, Argonne National Laboratory, Argonne, Illinois, USA; 5 Northwestern Synchrotron Research Center, Life Sciences Collaborative Access Team, Northwestern University, Argonne, Illinois, USA; 6 Department of Molecular Physiology and Biological Physics, University of Virginia, Charlottesville, Virginia, USA; 7 Department of Microbiology, Immunology and Infectious Diseases, University of Calgary, Calgary, Alberta, Canada; 8 Department of Biochemistry and Molecular Genetics, Feinberg School of Medicine, Northwestern University, Chicago, Illinois, USA; Indiana University, Bloomington, Indiana, USA

**Keywords:** protein structure-function, *Clostridium difficile*, structural genomics, pipeline, X-ray, Clostridiodes

## Abstract

*Clostridioides difficile* causes life-threatening gastrointestinal infections. It is a high-risk pathogen due to a lack of effective treatments, antimicrobial resistance, and a poorly conserved genomic core. Herein, we report 30 X-ray structures from a structure genomics pipeline spanning 13 years, representing 10.2% of the X-ray structures for this important pathogen.

## ANNOUNCEMENT

*Clostridioides difficile* is a Gram-positive, anaerobic, rod-shaped bacterium that infects humans via orofecal transmission (1, 2). The microbiome normally prevents germination and toxin production in the gut (3, 4). However, under dysbiosis, *C. difficile* spores germinate into the vegetative-toxin-producing form, leading to life-threatening diarrhea, gut perforation, and pseudomembranous colitis (5–7). The 2020 Annual Report for the Emerging Infections Program for *C. difficile* Infection (CDI) reports an incidence rate of 101.3 cases per 100,000 persons (8). Unfortunately, only three antimicrobials (metronidazole, vancomycin, and fidaxomicin) and microbiome transplants are available to treat CDI, but these show reduced effectiveness due to increasing antimicrobial resistance (8–12). The discovery of new approaches to treat CDI is urgent. However, novel drug discovery is hindered by the low-conserved genomic core of *C. difficile* and limited knowledge about this organism (13). The Center for Structural Biology of Infectious Diseases (CSBID, formerly the Center for Structural Genomics of Infectious Diseases) implemented a structural biology pipeline starting in 2010 through paired efforts with systems biology, bioinformatics, and other research specialists in *C. difficile* pathogenesis. This project ultimately involved 335 proteins linked with pathogenesis, metabolism, and antimicrobial resistance. These structures could serve as a resource to increase our understanding of *C. difficile* pathogenesis as well as essential proteins for effective drug or vaccine development. At the time the CSBID started the structural genomics pipeline in 2010, only 26 structures were deposited in the Research Collaboratory for Structural Bioinformatics Protein Data Bank (PDB; https://www.rcsb.org/). Our main bottleneck in *C. difficile* structure determination early in the project was protein expression, followed by solubility. Hence, we implemented a second pipeline of 79 targets using genes codon-optimized for expression in *Escherichia coli*, new constructs of soluble domains, or cloned in the vector pCPD to improve solubility. Nonetheless, more than 50% of the targets failed at expression and solubility.

**Fig 1:**
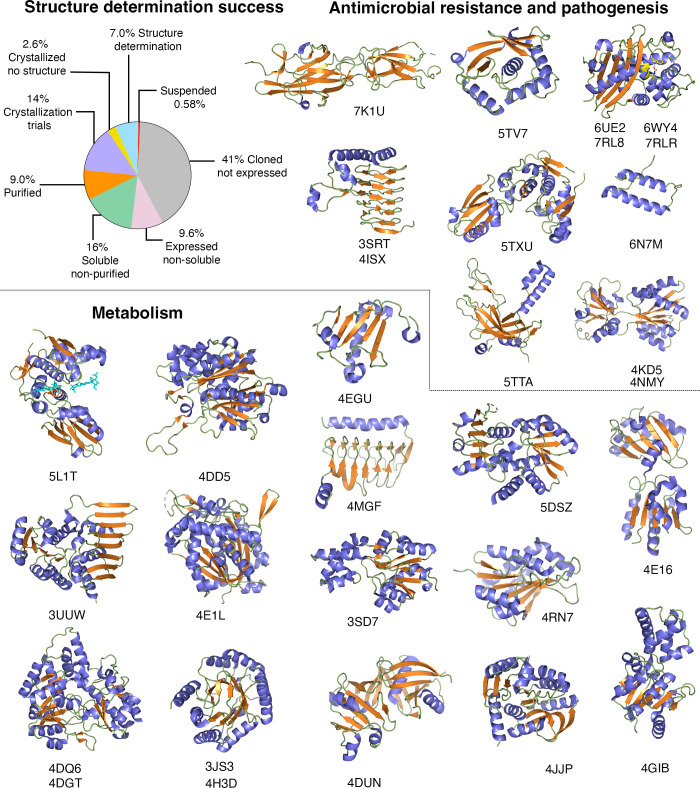
*C. difficile* proteins in the CSBID pipeline and representative X-ray structures. The chart shows the success rate of proteins in the CSGID pipeline from a total of 335 targets on both the 2010 and 2018 structure determination pipelines. Twenty unique structures are represented as cartoons: β-sheets are depicted in orange, α-helices in blue, and loops in green. Associated variants or structures in complexes with ligands are annotated below each unique structure, which makes a total of 30 structures. The proteins were classified according to their function if known in pathogenic behavior, antimicrobial resistance, and metabolism. Protein names, associated ligands, and crystallography details are described in Table 1.

The overall success of structure determination for *C. difficile* in both CSBID pipelines was 7%, with a total of 23 unique structures and eight variants or ligand-bound proteins determined. From these structures, 16.2% were codon-optimized and cloned into the pCPD vector (14). The structures presented here are part of the *C. difficile* 630, R20291, and Y384 sets nominated at CSBID (Fig. 1, Table 1). Of the 31 structures determined, 30 are reported here, with one structure published separately (15). In total, these structures represent 10.2% of the 295 protein structures for *C. difficile* currently in the database.

**TABLE 1:** Data quality and refinement statistics^*a*^

PDB accession code	3JS3	4H3D	3UUW
Name	Type I 3-dehydroquinate dehydratase (AroD) with 3-amino-4,5-dihydroxy-cyclohex-1 enecarboxylate bound	Type I 3-dehydroquinate dehydratase (AroD) with covalent modified comenic acid bound	Dehydrogenase (MviM)
Data collection			
Space group	*P*1211	*P*12_1_1	*P*1
*a, b, c* (Å)	60.47, 139.62, 66.77	60.27, 138.69, 66.31	69.52, 69.53, 83.09
*α, β, γ (º)*	90.00, 90.63, 90.00	90.00, 90.01, 90.00	98.10, 106.97, 115.73
Resolution range (Å)	30.00–2.20 (2.24–2.20)	30.00–1.95 (1.98–1.95)	30.00–1.63 (1.66–1.63)
No. of reflections	55,938 (2,854)	79,221 (3,903)	155,282 (7,631)
*R*_merge_ (%)	6.6 (61.4)	6.7 (45.8)	6.5 (57.8)
Completeness (%)	99.9 (100.0)	99.8 (99.6)	97.1 (94.8)
[I/σ(I)]	17.3 (2.2)	17.4 (3.1)	16.7 (2.7)
Multiplicity	3.8 (3.8)	3.8 (3.8)	4.3 (4.2)
Wilson B factor (Å2)	44.6	24.7	22.3
Refinement			
Resolution range (Å)	29.55–2.20 (2.26–2.20)	27.64–1.95 (2.00–1.95)	29.97–1.63 (1.67–1.63)
Completeness (%)	99.8 (98.0)	99.8 (99.1)	97.1 (95.3)
No. of reflections	55,907 (4,028)	78,860 (5,731)	154,394 (11,159)
*R*_work_/*R*_free_, (%)	18.9/24.1 (28.0/33.0)	15.6/19.9 (18.7/23.1)	15.8/19.5 (23.0/28.5)
Protein chains/atoms	4/8,036	4/8,036	4/9,654
Ligand/Solvent atoms	44/318	94/817	91/1191
Mean temperature factor (Å^2^)	21.9	24.4	18.7
Coordinate deviations			
R.m.s.d. bonds (Å)	0.012	0.013	0.011
R.m.s.d. angles (º)	1.537	1.325	1.351
Ramachandran plot			
Favored (%)	97.0	99.0	98.0
Allowed (%)	2.0	1.0	2.0
Outside allowed (%)	1.0	0.0	0.0

PDB accession code	4DD5	4DQ6	4DGT
Name	Biosynthetic thiolase (ThlA1)	Putative aminotransferase with PLP-bound	Putative aminotransferase with PLP-bound with magnesium formate
Data collection			
Space group	*I*222	*P*12_1_1	*P*12_1_1
*a, b, c* (Å)	58.85, 107.41, 151.31	79.42, 56.09, 85.27	78.74, 55.62, 79.13
*α, β, γ (º)*	90.00, 90.00, 90.00	90.00, 99.33, 90.00	90.00, 91.25, 90.00
Resolution range (Å)	30.00–1.25 (1.27–1.25)	50.00–1.50 (1.53–1.50)	50.00–1.55 (1.58–1.55)
No. of reflections	131,046 (6,295)	119,055 (5,559)	99,598 (4,608)
*R*_merge_ (%)	5.0 (57.0)	7.2 (49.3)	7.4 (40.7)
Completeness (%)	99.7 (96.8)	99.5 (93.6)	99.5 (94.1)
[I/σ(I)]	23.4 (2.2)	21.5 (2.3)	19.0 (2.3)
Multiplicity	5.0 (4.6)	4.0 (2.3)	3.6 (2.5)
Wilson B factor (Å^2^)	17.4	15.9	13.0
Refinement			
Resolution range (Å)	29.13–1.25 (1.28–1.25)	50.00–1.50 (1.54–1.50)	50.00–1.55 (1.59–1.55)
Completeness (%)	99.2 (90.7)	99.5 (94.9)	99.5 (95.9)
No. of reflections	131,041 (8,476)	118,099 (8,034)	99,012 (6,850)
*R*_work_/*R*_free_, (%)	11.7/13.5 (23.9/24.4)	15.3/18.6 (25.1/27.6)	14.7/18.1(19.9/23.4)
Protein chains/atoms	1/2,809	2/6,258	2/6,274
Ligand/Solvent atoms	508	33/1032	43/878
Mean temperature factor (Å^2^)	12.8	22.3	17.2
Coordinate deviations			
R.m.s.d. bonds (Å)	0.011	0.017	0.017
R.m.s.d. angles (º)	1.563	1.815	1.817
Ramachandran plot			
Favored (%)	99.0	97.0	97.0
Allowed (%)	1.0	3.0	3.0
Outside allowed (%)	0.0	0.0	0.0

PDB accession code	3SD7	4DUN	4E1L
Name	Putative phosphatase	Putative phenazine biosynthesis PhzC/PhzF	Acetoacetyl-CoA thiolase (ThlA2)
Data collection			
Space group	*I*41	*P*3_1_21	P12_1_1
*a, b, c* (Å)	116.33, 116.33, 36.08	63.65, 63.65, 135.12	103.32, 78.92, 127.03
*α, β, γ (º)*	90.00, 90.00, 90.00	90.00, 90.00, 120.00	90.00, 112.73, 90.00
Resolution range (Å)	30.00–1.70 (1.73–1.70)	51.04–1.76 (1.81–1.76)	39.20–2.00 (2.07–2.00)
No. of reflections	26,648 (1,216)	32,236 (2,336)	126,930 (12,004)
*R*_merge_ (%)	4.8 (54.1)	9.3 (68.4)	7.6 (62.2)
Completeness (%)	99.2 (91.1)	99.9 (100.0)	99.1 (95.0)
[I/σ(I)]	35.0 (2.8)	9.9 (2.1)	16.3 (2.2)
Multiplicity	7.2 (5.3)	5.4 (5.6)	4.9 (3.9)
Wilson B factor (Å^2^)	29.7	25.7	30.6
Refinement			
Resolution range (Å)	29.65–1.70 (1.75–1.70)	23.12–1.76 (1.81–1.76)	39.05–2.00 (2.07–2.00)
Completeness (%)	99.1 (91.1)	99.9 (100.0)	98.8 (91.9)
No. of reflections	26,643 (1,794)	33,802 (2,927)	132,020 (11,649)
*R*_work_/*R*_free_, (%)	16.7/19.3 (28.6/40.7)	15.8/19.0 (20.3/24.9)	17.5/20.7 (23.5/26.9)
Protein chains/atoms	1/1,759	1/2,041	4/11,031
Ligand/Solvent atoms	24/161	34/370	57/871
Mean temperature factor (Å^2^)	39.3	30.2	40.9
Coordinate deviations			
R.m.s.d. bonds (Å)	0.013	0.010	0.008
R.m.s.d. angles (º)	1.298	1.090	1.090
Ramachandran plot			
Favored (%)	97.0	97.0	99.0
Allowed (%)	3.0	3.0	1.0
Outside allowed (%)	0.0	0.0	0.0

PDB accession code	4EGU	4GIB	4MFG
Name	Histidine triad nucleotide-binding protein	β-Phosphoglucomutase (PgmB)	Putative carbonic anhydrase
Data collection			
Space group	*P*2_1_2_1_2_1_	*P*12_1_1	*P*6_3_
*a, b, c* (Å)	38.06, 65.85, 89.04	61.80, 51.36, 79.44	127.09, 127.09, 76.732
*α, β, γ (º)*	90.00, 90.00, 90.00	90.00, 112.04, 90.00	90.00, 90.00, 120.00
Resolution range (Å)	30.00–0.95 (0.98–0.95)	30.00–2.27 (2.31–2.27)	30.00–2.00 (2.03–2.00)
No. of reflections	140,927 (13,024)	21,526 (1,055)	47,926 (2,398)
*R*_merge_ (%)	5.3 (48.6)	9.7 (43.9)	10.7 (61.0)
Completeness (%)	99.3 (93.5.0)	99.3 (97.4)	99.9 (100.0)
[I/σ(I)]	27.1 (2.2)	11.7 (2.6)	29.5 (3.8)
Multiplicity	5.8 (3.5)	3.6 (3.3)	9.7 (9.5)
Wilson B factor (Å^2^)	11.1	35.4	19.1
Refinement			
Resolution range (Å)	32.90–0.95 (0.98–0.95)	29.92–2.27 (2.33–2.27)	29.36–2.00 (2.05–2.00)
Completeness (%)	98.1 (90.1)	99.2 (96.0)	99.9 (100.0)
No. of reflections	141,350 (12,585)	21,442 (1,509)	47,715 (3,515)
*R*_work_/*R*_free_, (%)	13.6/15.2 (27.4/28.0)	19.3/25.9 (22.7/34.0)	21.2/26.7.5 (29.6/31.6)
Protein chains/atoms	2/1,851	2/3,546	4/5,152
Ligand/Solvent atoms	52/345	34/170	6/406
Mean temperature factor (Å^2^)	17.3	26.3	42.27
Coordinate deviations			
R.m.s.d. bonds (Å)	0.010	0.011	0.012
R.m.s.d. angles (º)	1.577	1.252	1.619
Ramachandran plot			
Favored (%)	100.0	97.0	96.0
Allowed (%)	0.0	3.0	4.0
Outside allowed (%)	0.0	0.0	0.0

PDB accession code	4JJP	4E16	5DZS
Name	Phosphomethylpyrimidine kinase (ThiD)	Precorrin-4-C-(11)-methyltransferase	Shikimate dehydrogenase 1
Data collection			
Space group	*P*2_1_2_1_2_1_	*P*4_3_2_1_2	*P*_1_
*a, b, c* (Å)	56.75, 79.74, 92.86	60.24, 60.24, 147.95	41.22, 47.05, 74.74
*α, β, γ (º)*	90.00, 90.00, 90.00	90.00, 90.00, 90.00	77.98, 76.44, 89.09
Resolution range (Å)	30.00–2.05 (2.09–2.05)	55.79–2.49 (2.53–2.49)	30.00–1.50 (1.53–1.50)
No. of reflections	26,751 (1,321)	10,113 (477)	82,004 (-)
*R*_merge_ (%)	7.7 (57.5)	9.8 (92.4)	3.8 (45.4)
Completeness (%)	99.9 (99.8)	99.3 (97.1)	96.1 (94.5)
[I/σ(I)]	21.7 (3.1)	11.6 (2.6)	29.0 (3.1)
Multiplicity	6.0 (5.9)	12.1 (8.9)	3.9 (4.0)
Wilson B factor (Å^2^)	33.0	82.0	23.6
Refinement			
Resolution range (Å)	28.86–2.06 (2.11–2.06)	41.00–2.49 (2.56–2.49)	29.70–1.50 (1.54–1.50)
Completeness (%)	99.3 (91.6)	99.0 (92.8)	95.9 (93.2)
No. of reflections	26,704 (1,805)	10,064 (582)	81,997 (5,893)
*R*_work_/*R*_free_, (%)	18.1/21.6 (25.2/26.3)	20.4/25.8 (41.7/52.2)	16.3/18.5 (22.9/26.5)
Protein chains/atoms	2/3,427	1/1,773	2/4,200
Ligand/Solvent atoms	15/147	-	15/423
Mean temperature factor (Å^2^)	35.4	58.4.2	38.5
Coordinate deviations			
R.m.s.d. bonds (Å)	0.013	0.015	0.009
R.m.s.d. angles (º)	1.571	1.983	1.373
Ramachandran plot			
Favored (%)	98.0	92.0	97.0
Allowed (%)	2.0	5.0	2.0
Outside allowed (%)	0.0	2.0	1.0

PDB accession code	4RN7	3SRT	4ISX
Name	*N*-acetylmuramoyl-l-alanine amidase	Maltose-*O*-acetyltransferase	Maltose-*O*-acetyltransferase with acetyl-CoA bound
Data collection			
Space group	*P*3_2_21	*P*2_1_3	*F*23
*a, b, c* (Å)	66.41, 66.41, 96.2	152.88, 152.88, 152.88	167.89, 167.89, 167.89
*α, β, γ (º)*	90.00, 90.00, 120.00	90.00, 90.01, 90.00	90.00, 90.00, 90.00
Resolution range (Å)	28.80–1.71 (1.75–1.72)	48.50–2.50 (2.56–2.50)	39.00–2.70 (2.76–2.70)
No. of reflections	26,769 (1,344)	41,225 (2,058)	10,868 (722)
*R*_merge_ (%)	5.5 (71.7)	9.8 (75.7)	8.3 (67.1)
Completeness (%)	99.8 (100.0)	99.9 (99.9)	99.9 (100.0)
[I/σ(I)]	54.7 (3.3)	29 (2.6)	36.9 (3.3)
Multiplicity	8.3 (8.4)	7.1 (6.9)	7.4 (7.5)
Wilson B factor (Å^2^)	27.4	47.1	66.6
Refinement			
Resolution range (Å)	28.76–1.72 (1.78–1.72)	48.35–2.50 (2.50–0.00)	38.52–2.70 (2.97–2.70)
Completeness (%)	99.7 (100.0)	96.3 (88.0)	99.8 (100.0)
No. of reflections	28,040 (0)	41,673 (2,083)	11,383 (547)
*R*_work_/*R*_free_, (%)	19.5/21.5 (24.7/28.4)	16.4/18.6 (23.7/27.4)	19.4/25.2.5 (27.0/36.0)
Protein chains/atoms	1/1,426	2/2,926	2/2,913
Ligand/Solvent atoms	29/123	96/99	28/114
Mean temperature factor (Å^2^)	42.2	48.9	62.1
Coordinate deviations			
R.m.s.d. bonds (Å)	0.006	0.009	0.003
R.m.s.d. angles (º)	0.998	1.131	0.715
Ramachandran plot			
Favored (%)	95.0	98.0	98.0
Allowed (%)	5.0	2.0	2.0
Outside allowed (%)	0.0	0.0	0.0

PDB accession code	6UE2	6WY4	7RL8
Name	Class D β-lactamase	Class D β-lactamase WT	Class D β-lactamase C79A variant
Data collection			
Space group	*P*2_1_2_1_2_1_	*P*2_1_2_1_2_1_	*P*2_1_2_1_2_1_
*a, b, c* (Å)	84.98, 93.20, 137.58	85.33, 93.26, 138.05	86.12, 93.87, 138.34
*α, β, γ (º)*	90.00, 90.00, 90.00	90.00, 90.00, 90.00	90.00, 90.00, 90.00
Resolution range (Å)	30.00–1.85 (1.88–1.85)	30.00–1.80 (1.83–1.80)	30.00–1.95 (1.98–1.95)
No. of reflections	93,218 (4,591)	102,923 (5,107)	82,125 (4,066)
*R*_merge_ (%)	7.4 (79.4)	7.0 (79.3)	7.4 (72.2)
Completeness (%)	99.9 (99.2)	100.0 (100.0)	100.0 (100.0)
[I/σ(I)]	25.6 (2.3)	24.6 (2.5)	25.1 (2.9)
Multiplicity	7.1 (5.9)	6.2 (6.2)	7.4 (7.4)
Wilson B factor (Å^2^)	26.0	23.7	31.9
Refinement			
Resolution range (Å)	29.56–1.85 (1.90–1.85)	29.66–1.80 (1.85–1.80)	29.84–1.95 (2.00–1.95)
Completeness (%)	99.6 (95.2)	100.0 (100.0)	99.8 (98.9)
No. of reflections	93,127 (6,518)	102,485 (7,457)	82,052 (5,929)
*R*_work_/*R*_free_, (%)	16.5/19.7 (26.1/26.2)	17.6/20.9 (24.7/25.2)	17.8/20.7 (23.6/26.5)
Protein chains/atoms	4/8,153	4/8,127	4/8,130
Ligand/Solvent atoms	28/881	8/968	17/730
Mean temperature factor (Å^2^)	34.5	30.9	39.8
Coordinate deviations			
R.m.s.d. bonds (Å)	0.006	0.004	0.004
R.m.s.d. angles (º)	1428	1.234	1.242
Ramachandran plot			
Favored (%)	97.0	97.0	97.0
Allowed (%)	3.0	3.0	3.0
Outside allowed (%)	0.0	0.0	0.0

PDB accession code	7RLR	4KD5	4NMY
Name	Class D β-lactamase K83A variant	Substrate binding domain of putative molybdenum ABC transporter	Substrate-binding protein of ABC transporter with thiamin-bound
Data collection			
Space group	*P*2_1_2_1_2_1_	*C*2_2_2_1_	*P*2_1_2_1_2_1_
*a, b, c* (Å)	85.53, 93.62, 138.06	123.64, 141.07, 122.59	49.53, 76.86, 154.90
*α, β, γ (º)*	90.00, 90.00, 90.00	90.00, 90.00, 90.00	90.00, 90.00, 90.00
Resolution range (Å)	30.00–1.88 (1.91–1.88)	50.00–2.49 (2.54–2.49)	50.00–1.90 (1.93–1.90)
No. of reflections	90,491 (4,507)	37,512 (1,831)	47,478 (2,273)
*R*_merge_ (%)	6.8 (79.0)	8.0 (78.2)	8.5 (58.1)
Completeness (%)	100.0 (100.0)	99.6 (99.8)	99.7 (98.4)
[I/σ(I)]	26.7 (2.5)	15.3 (2.0)	8.5 (2.5)
Multiplicity	6.9 (7.0)	5.4 (4.3)	4.3 (3.5)
Wilson B factor (Å^2^)	26.8	58.3	19.3
Refinement			
Resolution range (Å)	29.71–1.88 (1.93–1.88)	34.09–2.50 (2.56–2.50)	38.43–1.90 (1.94–1.90)
Completeness (%)	99.8 (97.9)	97.0 (89.0)	98.2 (80.0)
No. of reflections	90,410 (6,449)	38,192 (2,355)	49,302 (2,207)
*R*_work_/*R*_free_, (%)	16.8/19.8 (22.8/26.1)	20.4/25.9 (31.4/37.1)	16.4/20.3 (21.8/26.7)
Protein chains/atoms	4/8,114	4/7,032	2/4,766
Ligand/Solvent atoms	33/886	34/82	36/419
Mean temperature factor (Å^2^)	33.1	73.6	21.4
Coordinate deviations			
R.m.s.d. bonds (Å)	0.005	0.004	0.008
R.m.s.d. angles (º)	1.261	0.789	1.097
Ramachandran plot			
Favored (%)	97	98.0	99
Allowed (%)	3	2.0	1
Outside allowed (%)	0	0.0	0

PDB accession code	7 K1U	6 N7M	5TTA
Name	SrtB-anchored collagen-binding adhesin fragment	Hypothetical protein CD630_05490	Hypothetical protein CD630_21270
Data collection			
Space group	*P*3_1_2_1_	*P*1_2_1_1_	*P*1
*a, b, c* (Å)	69.32, 69.32, 185.53	26.62, 58.69, 77.33	30.78, 51.04, 81.12
*α, β, γ (º)*	90.00, 90.00, 120.00	90.00, 94.47, 120.00	73.38, 82.06, 86.35
Resolution range (Å)	30.00–2.40 (2.44–2.40)	46.70–1.78 (1.81–1.78)	30.47–1.85 (1.90–1.85)
No. of reflections	21,161 (1,018)	22,345 (1,068)	39,052 (1,460)
*R*_merge_ (%)	9.0 (83.4)	10.2 (83.0)	7.1 (61.4)
Completeness (%)	100.0 (100.0)	97.7 (97.9)	97.6 (95.6)
[I/σ(I)]	22.0 (1.8)	11.1 (2.1)	18.7 (3.3)
Multiplicity	7.2 (7.4)	5.2 (4.1)	7.8 (7.7)
Wilson B factor (Å^2^)	49.7	25.2	25.9
Refinement			
Resolution range (Å)	29.64–2.40 (2.46–2.40)	24.52–1.78 (1.83–1.78)	30.48–1.85 (1.90–1.85)
Completeness (%)	99.8 (99.4)	97.7 (98.5)	88.6 (51.0)
No. of reflections	21,107 (1,481)	22,315 (1,612)	37,350 (1,402)
*R*_work_/*R*_free_, (%)	17.4/21.7 (25.7/28.4)	24.6/28.4 (31.9/39.1)	18.0/22.9 (30.0/30.3)
Protein chains/atoms	1/1,945	4/2,049	2/3,434
Ligand/Solvent atoms	14/156	213	327
Mean temperature factor (Å^2^)	52.2	29.8	43.2
Coordinate deviations			
R.m.s.d. bonds (Å)	0.006	0.008	0.008
R.m.s.d. angles (º)	1.426	1.456	0.806
Ramachandran plot			
Favored (%)	98	97.0	99.0
Allowed (%)	2	3.0	1.0
Outside allowed (%)	0	0.0	0.0

PDB accession code	5I1T	5TXU	5TV7
Name	Stage II sporulation protein D (SpoIID) with triacetylchitotriose bound	Stage II sporulation protein D (SpoIID)	Peptidoglycan-binding protein with glutamine hydroxamate bound
Data collection			
Space group	*P*3_1_2_1_	*P*3_1_2_1_	*C*121
*a, b, c* (Å)	98.08, 98.08, 108.32	98.04, 98.04, 108.24	134.95, 35.01, 101.53
*α, β, γ (º)*	90.00, 90.00, 120.00	90.00, 90.00, 120.00	90.00, 126.25, 90.00
Resolution range (Å)	30.00–2.60 (2.64–2.60)	30.00–1.95 (1.98–1.95)	29.08–2.05 (2.09–2.05)
No. of reflections	18,969 (−)	44,267 (−)	24,487 (1,195)
Rmerge (%)	6.7 (60.8)	13.0 (50.8)	9.3 (76.9)
Completeness (%)	99.9 (100.0)	100.0 (100.0)	100.0 (99.9)
[I/σ(I)]	30.8 (4.1)	15.4 (5.1)	28.1 (2.0)
Multiplicity	8.8 (9.3)	9.3 (9.4)	5.0 (5.0)
Wilson B factor (Å^2^)	69.1	34.1	32.2
Refinement			
Resolution range (Å)	29.08–2.60 (2.67–2.60)	29.06–1.95 (2.00–1.95)	29.08–2.05 (2.10–2.05)
Completeness (%)	99.8 (99.7)	99.9 (99.6)	99.8 (98.1)
No. of reflections	18,943 (1,362)	44,232 (3,227)	24,464 (1,715)
*R*_work_/*R*_free_, (%)	15.5/19.6 (20.0/30.8)	15.9/18.4 (18.8/24.2)	17.2/21.0 (27.9/27.4)
Protein chains/atoms	1/2,280	1/2,280	2/2,460
Ligand/Solvent atoms	95/147	72/328	22/201
Mean temperature factor (Å^2^)	69.2	44.3	48.2
Coordinate deviations			
R.m.s.d. bonds (Å)	0.011	0.009	0.008
R.m.s.d. angles (º)	1.477	1.372	1.313
Ramachandran plot			
Favored (%)	97.0	97.0	98.0
Allowed (%)	3.0	3.0	2.0
Outside allowed (%)	0.0	0.0	0.0

^*a*^ Values in parentheses are for the outer shell.

Some genes from *C. difficile* were cloned using the specific primers for each gene into the pMCSG53 (16) and pMCSG7 (17) vectors. Other genes were synthesized and codon-optimized for expression in *E. coli* and cloned into pCPD (14) or pMCSG53 (16) vectors by Twist Biosciences (San Francisco, CA). The plasmids were transformed in *E. coli* BL21(DE3)(pMagic) or BL21-CodonPlus(DE3)-RIL and tested for expression and solubility (18). Proteins were expressed, purified (19), tag-cleaved, and set up in crystallization trials (20, 21). Protein crystals were cryoprotected and flash-frozen for X-ray diffraction. Data were collected at the Advanced Photon Source at Argonne National Laboratories (Table 1). The structures of the proteins with <30% structural homology were determined by single anomalous diffraction (SAD) using the automatic structure solution HKL-3000 (22) and the auto-build package [PHENIX V 1.13 (23)]. The structure of native-expressed proteins was determined by molecular replacement [PHASER (24), MORDA, and MRBUMP from the CCP4 suite V 7.0.044 (25)]. For structure refinement, REFMAC5 V 5.5.0102–5.8.0258 (26) or PHENIX V 1.13 were used and corrected in Coot (27).

Water molecules were generated using ARP/wARP (28), and ligands were fit into electron density maps in Coot. Translation-Libration-Screw (TLS) groups were generated by the TLS motion determination (TLSMD) server (29), and corrections were applied at the final steps of refinement. Models were validated using MolProbity (30), and the coordinates of the final models and experimental data were deposited in the PDB.

## Data Availability

The details of each structure, such as constructs, primers for cloning, status, purification, crystallization, and PDB codes, are available online at CSBID.org, listed as *Clostridium difficile* 630, *Peptoclostridum difficile*, and *Clostridium difficile*. Requests for specific methods can be sent to the Center for Structural Biology of Infectious Diseases. All coordinates for all final models, experimental data, and software versions have been deposited to PDB with accession codes: 3JS3, 3SD7, 3SRT, 3UUW, 4DD5, 4DGT, 4DQ6, 4DUN, 4E16, 4EGU, 4E1L, 4GIB, 4H3D, 4ISX, 4JJP, 4KD5, 4MFG, 4NMY, 4RN7, 5DZS, 5I1T, 5TTA, 5TV7, 5TXU, 6N7M, 6UE2, 6WY4, 7K1U, 7RL8, 7RLR.

## References

[1] Burke KE, Lamont JT. 2014. Clostridium difficile infection: a worldwide disease. Gut Liver 8:1–6. doi:10.5009/gnl.2014.8.1.1 24516694 PMC3916678

[2] Sandhu BK, McBride SM. 2018. Clostridioides difficile. Trends Microbiol 26:1049–1050. doi:10.1016/j.tim.2018.09.004 30297117 PMC6637408

[3] Theriot C.M, Young VB. 2014. Microbial and metabolic interactions between the gastrointestinal tract and Clostridium difficile infection. Gut Microbes 5:86–95. doi:10.4161/gmic.27131 24335555 PMC4049944

[4] Abt MC, McKenney PT, Pamer EG. 2016. Clostridium difficile colitis: pathogenesis and host defence. Nat Rev Microbiol 14:609–620. doi:10.1038/nrmicro.2016.108 27573580 PMC5109054

[5] Theriot Casey M, Koenigsknecht MJ, Carlson PE Jr, Hatton GE, Nelson AM, Li B, Huffnagle GB, Z Li J, Young VB. 2014. Antibiotic-induced shifts in the mouse gut microbiome and metabolome increase susceptibility to Clostridium difficile infection. Nat Commun 5:3114. doi:10.1038/ncomms4114 24445449 PMC3950275

[6] Chandrasekaran R, Lacy DB. 2017. The role of toxins in Clostridium difficile infection. FEMS Microbiol Rev 41:723–750. doi:10.1093/femsre/fux048 29048477 PMC5812492

[7] Kordus SL, Thomas AK, Lacy DB. 2022. Clostridioides difficile toxins: mechanisms of action and antitoxin therapeutics. Nat Rev Microbiol 20:285–298. doi:10.1038/s41579-021-00660-2 34837014 PMC9018519

[8] U.S. Department of Health and Human Services C. 2019. CDC. Antibiotic resistance threats in the United States

[9] Dieterle MG, Rao K, Young VB. 2019. Novel therapies and preventative strategies for primary and recurrent Clostridium difficile infections. Ann N Y Acad Sci 1435:110–138. doi:10.1111/nyas.13958 30238983 PMC6312459

[10] Feuerstadt P, Louie TJ, Lashner B, Wang EEL, Diao L, Bryant JA, Sims M, Kraft CS, Cohen SH, Berenson CS, Korman LY, Ford CB, Litcofsky KD, Lombardo M-J, Wortman JR, Wu H, Auniņš JG, McChalicher CWJ, Winkler JA, McGovern BH, Trucksis M, Henn MR, von Moltke L. 2022. SER-109, an oral microbiome therapy for recurrent Clostridioides difficile infection. N Engl J Med 386:220–229. doi:10.1056/NEJMoa2106516 35045228

[11] Gilbert JA. 2022. Microbiome therapy for recurrent Clostridioides difficile. Lancet Microbe 3:e334. doi:10.1016/S2666-5247(22)00096-9 35544093

[12] Revolinski SL, Munoz-Price LS. 2019. Clostridium difficile in immunocompromised hosts: a review of epidemiology, risk factors, treatment, and prevention. Clin Infect Dis 68:2144–2153. doi:10.1093/cid/ciy845 30281082

[13] Norsigian CJ, Danhof HA, Brand CK, Midani FS, Broddrick JT, Savidge TC, Britton RA, Palsson BO, Spinler JK, Monk JM. 2022. Systems biology approach to functionally assess the Clostridioides difficile pangenome reveals genetic diversity with discriminatory power. Proc Natl Acad Sci U S A 119:e2119396119. doi:10.1073/pnas.2119396119 35476524 PMC9170149

[14] Biancucci M, Dolores JS, Wong J, Grimshaw S, Anderson WF, Satchell KJF, Kwon K. 2017. New ligation independent cloning vectors for expression of recombinant proteins with a self-cleaving CPD/6XHis-tag. BMC Biotechnol 17:1. doi:10.1186/s12896-016-0323-4 28056928 PMC5216533

[15] Alexander LT, Durairaj J, Kryshtafovych A, Abriata LA, Bayo Y, Bhabha G, Breyton C, Caulton SG, Chen J, Degroux S, Ekiert DC, Erlandsen BS, Freddolino PL, Gilzer D, Greening C, Grimes JM, Grinter R, Gurusaran M, Hartmann MD, Hitchman CJ, Keown JR, Kropp A, Kursula P, Lovering AL, Lemaitre B, Lia A, Liu S, Logotheti M, Lu S, Markússon S, Miller MD, Minasov G, Niemann HH, Opazo F, Phillips GN, Davies OR, Rommelaere S, Rosas-Lemus M, Roversi P, Satchell K, Smith N, Wilson MA, Wu K-L, Xia X, Xiao H, Zhang W, Zhou ZH, Fidelis K, Topf M, Moult J, Schwede T. 2023. Protein target highlights in CASP15: analysis of models by structure providers. Proteins. doi:10.1002/prot.26545 PMC10792529 37493353

[16] Eschenfeldt WH, Makowska-Grzyska M, Stols L, Donnelly MI, Jedrzejczak R, Joachimiak A. 2013. New LIC vectors for production of proteins from genes containing rare codons. J Struct Funct Genomics 14:135–144. doi:10.1007/s10969-013-9163-9 24057978 PMC3933008

[17] Eschenfeldt WH, Lucy S, Millard CS, Joachimiak A, Mark ID. 2009. A family of LIC vectors for high-throughput cloning and purification of proteins. Methods Mol Biol 498:105–115. doi:10.1007/978-1-59745-196-3_7 18988021 PMC2771622

[18] Kwon K, Peterson SN. 2014. High-throughput cloning for biophysical applications, p 61–74. In Anderson WF (ed), Structural genomics and drug discovery: methods and protocols. Springer, New York, NY. doi:10.1007/978-1-4939-0354-2 24590709

[19] Shuvalova L. 2014. Parallel protein purification. Methods Mol Biol 1140:137–143. doi:10.1007/978-1-4939-0354-2_10 24590714

[20] Skarina T, Xu X, Evdokimova E, Savchenko A. 2014. High-throughput crystallization screening. Methods Mol Biol 1140:159–168. doi:10.1007/978-1-4939-0354-2_12 24590716

[21] Wu N, Christendat D, Dharamsi A, Pai EF. 2000. Purification, crystallization and preliminary X-ray study of orotidine 5'-monophosphate decarboxylase. Acta Crystallogr D Biol Crystallogr 56:912–914. doi:10.1107/s090744490000576x 10930842

[22] Minor W, Cymborowski M, Otwinowski Z, Chruszcz M. 2006. HKL-3000: the integration of data reduction and structure solution--from diffraction images to an initial model in minutes. Acta Crystallogr D Biol Crystallogr 62:859–866. doi:10.1107/S0907444906019949 16855301

[23] Liebschner D, Afonine PV, Baker ML, Bunkóczi G, Chen VB, Croll TI, Hintze B, Hung LW, Jain S, McCoy AJ, Moriarty NW, Oeffner RD, Poon BK, Prisant MG, Read RJ, Richardson JS, Richardson DC, Sammito MD, Sobolev OV, Stockwell DH, Terwilliger TC, Urzhumtsev AG, Videau LL, Williams CJ, Adams PD. 2019. Macromolecular structure determination using X-rays, neutrons and electrons: recent developments in Phenix. Acta Crystallogr D Struct Biol 75:861–877. doi:10.1107/S2059798319011471 31588918 PMC6778852

[24] McCoy AJ, Grosse-Kunstleve RW, Adams PD, Winn MD, Storoni LC, Read RJ. 2007. Phaser crystallographic software. J Appl Crystallogr 40:658–674. doi:10.1107/S0021889807021206 19461840 PMC2483472

[25] Winn MD, Ballard CC, Cowtan KD, Dodson EJ, Emsley P, Evans PR, Keegan RM, Krissinel EB, Leslie AGW, McCoy A, McNicholas SJ, Murshudov GN, Pannu NS, Potterton EA, Powell HR, Read RJ, Vagin A, Wilson KS. 2011. Overview of the CCP4 suite and current developments. Acta Crystallogr D Biol Crystallogr 67:235–242. doi:10.1107/S0907444910045749 21460441 PMC3069738

[26] Murshudov GN, Skubák P, Lebedev AA, Pannu NS, Steiner RA, Nicholls RA, Winn MD, Long F, Vagin AA. 2011. REFMAC5 for the refinement of macromolecular crystal structures. Acta Crystallogr D Biol Crystallogr 67:355–367. doi:10.1107/S0907444911001314 21460454 PMC3069751

[27] Emsley P, Cowtan K. 2004. Coot: model-building tools for molecular graphics. Acta Crystallogr D Biol Crystallogr 60:2126–2132. doi:10.1107/S0907444904019158 15572765

[28] Morris RJ, Perrakis A, Lamzin VS. 2003. ARP/wARP and automatic interpretation of protein electron density maps. Methods Enzymol 374:229–244. doi:10.1016/S0076-6879(03)74011-7 14696376

[29] Painter J, Merritt EA. 2006. Optimal description of a protein structure in terms of multiple groups undergoing TLS motion. Acta Crystallogr D Biol Crystallogr 62:439–450. doi:10.1107/S0907444906005270 16552146

[30] Chen VB, Arendall WB, Headd JJ, Keedy DA, Immormino RM, Kapral GJ, Murray LW, Richardson JS, Richardson DC. 2010. Molprobity: all-atom structure validation for macromolecular crystallography. Acta Crystallogr D Biol Crystallogr 66:12–21. doi:10.1107/S0907444909042073 20057044 PMC2803126

